# Activation of PPAR-α attenuates myocardial ischemia/reperfusion injury by inhibiting ferroptosis and mitochondrial injury via upregulating 14-3-3η

**DOI:** 10.1038/s41598-024-64638-9

**Published:** 2024-07-02

**Authors:** Tie Hu, Wen-peng Yu, Xiu-qi Wang, Zi-yao Wang, Zhi-qiang Xu, Fa-jia Hu, Ji-chun Liu, Fan Yu, Li-jun Wang

**Affiliations:** 1 Department of Cardiovascular Surgery, The Second Affiliated Hospital, Jiangxi Medical College, Nanchang University, Nanchang, 330006 Jiangxi China; 2 Department of Cardiovascular Surgery, The First Affiliated Hospital, Jiangxi Medical College, Nanchang University, Nanchang, 330006 Jiangxi China; 3 Department of Pathology, The First Affiliated Hospital of Gannan Medical University, Ganzhou, 341000 Jiangxi China

**Keywords:** PPAR-α, 14-3-3η, Myocardial ischemia/reperfusion injury, Ferroptosis, Cardioprotection, Cell biology, Genetics, Molecular biology, Biomarkers, Cardiology, Diseases, Health care, Molecular medicine, Pathogenesis

## Abstract

This study aimed to explore the effects of peroxisome proliferator-activated receptor α (PPAR-α), a known inhibitor of ferroptosis, in Myocardial ischemia/reperfusion injury (MIRI) and its related mechanisms. In vivo and in vitro MIRI models were established. Our results showed that activation of PPAR-α decreased the size of the myocardial infarct, maintained cardiac function, and decreased the serum contents of creatine kinase-MB (CK-MB), lactate dehydrogenase (LDH), and Fe^2+^ in ischemia/reperfusion (I/R)-treated mice. Additionally, the results of H&E staining, DHE staining, TUNEL staining, and transmission electron microscopy demonstrated that activation of PPAR-α inhibited MIRI-induced heart tissue and mitochondrial damage. It was also found that activation of PPAR-α attenuated MIRI-induced ferroptosis as shown by a reduction in malondialdehyde, total iron, and reactive oxygen species (ROS). In vitro experiments showed that intracellular contents of malondialdehyde, total iron, LDH, reactive oxygen species (ROS), lipid ROS, oxidized glutathione disulphide (GSSG), and Fe^2+^ were reduced by the activation of PPAR-α in H9c2 cells treated with anoxia/reoxygenation (A/R), while the cell viability and GSH were increased after PPAR-α activation. Additionally, changes in protein levels of the ferroptosis marker further confirmed the beneficial effects of PPAR-α activation on MIRI-induced ferroptosis. Moreover, the results of immunofluorescence and dual-luciferase reporter assay revealed that PPAR-α achieved its activity via binding to the 14-3-3η promoter, promoting its expression level. Moreover, the cardioprotective effects of PPAR-α could be canceled by pAd/14-3-3η-shRNA or Compound C11 (14-3-3η inhibitor). In conclusion, our results indicated that ferroptosis plays a key role in aggravating MIRI, and PPAR-α/14-3-3η pathway-mediated ferroptosis and mitochondrial injury might be an effective therapeutic target against MIRI.

## Introduction

Cardiovascular disease (CVD) is a major cause of disability and death worldwide, with acute myocardial infarction being a major type of CVD, posing a serious threat to human health^1,2^. Rebuilding coronary blood flow through thrombolysis, percutaneous coronary intervention, and vascular angioplasty remains the primary treatment for acute ischemic heart disease^3^. However, restoration of the coronary blood supply itself can also induce further death of myocardial cells and cause damage to myocardial tissue, thereby greatly reducing the clinical benefits of reperfusion for acute myocardial infarction (AMI) patients^4^. This phenomenon is also referred to myocardial ischemia/reperfusion injury (MIRI).

As a novel type of regulated cell death (RCD), ferroptosis has been reported to be involved in the pathological process of various diseases^5^. Ferroptosis exhibits distinct differences from other known RCDs. Biochemically, iron-dependent lipid peroxidation and depletion of intracellular glutathione (GSH) were the main signs of ferroptosis^6^. Morphologically, the main features of ferroptosis are reflected in mitochondrial shrinkage, reduction or disappearance of mitochondrial cristae, mitochondrial membrane wrinkling, and rupture of the outer membrane^7^. In previous studies, it has been found that ferroptosis participated in MIRI, and it has been identified as a potential diagnostic and therapeutic target for MIRI. Inhibiting ferroptosis also represents an effective approach to alleviate MIRI^8,9^.

As a member of the Ppar family, peroxisome proliferator-activated receptor alpha (PPAR-α) serves as a nuclear transcription factor involved in lipid metabolism^10,11^. PPAR-α can directly bind to specific PPAR-α response elements, regulating gene expression, and playing a crucial role in the regulation of oxidative stress^12,13^. Ppar family members include PPAR-α, Pparγ, and Pparβ, and the expression of these three subtypes varies in different tissues, with PPAR-α being most abundant in the heart^14^. Under normal conditions, PPAR-α upregulates the expression of triglyceride lipase (ATG1), mitochondrial lipid β-oxidation enzyme (CPT-1), and glucose oxidation negative regulator (PDK4) in myocardial cells, promoting fatty acid oxidation in myocardial cells and reducing the utilization of glucose, thus maintaining the energy balance of myocardial cells and meeting the physiological needs of the organism. In pathological conditions, PPAR-α has been shown to exert important biological effects, but its underlying mechanism involved remains unclear^14^. Previous studies have suggested that the protein level of PPAR-α is related to various cardiovascular diseases, such as hypertrophic cardiomyopathy, MIRI, and others^15^. In recent years, PPAR-α has regained widespread attention as a ferroptosis inhibitor in liver-related studies^16^. However, no reports on the role of PPAR-α in MIRI-induced ferroptosis are available.

The 14-3-3 protein family is involved in the stress response of various cells, including cardiomyocytes^17^. This protein family consists of seven isoforms (β, ε, η, γ, τ, σ, and ζ), each with specific expression patterns in both tissues and cells^18^. 14-3-3η stands out because of its prominent role in myocardial injury, not only participating in myocardial metabolism but also increasing its expression under stress to maintain stability of the endoplasmic reticulum and to alleviate myocardial cell damage^19,20^. Nevertheless, further attention needs to be paid to the underlying protective mechanisms of 14-3-3η in the myocardium.

In general, the following scientific hypothesis is proposed: PPAR-α can attenuate MIRI by nuclear translocation and binding to the 14-3-3η promoter sequence to activate 14-3-3η expression. This process transmits nuclear signals to the cytoplasm, attenuates mitochondrial injury, reduces oxidative stress levels, prevents intracellular iron overload in cardiomyocytes, and ultimately alleviates MIRI by targeting ferroptosis.

## Materials and methods

### Reagents and animals

The selective PPAR-α inhibitor (GW6471), PPAR-α agonist (GW7647), 14-3-3η inhibitor (Compound C11), and ferroptosis inhibitor (Ferrostatin-1, Fer-1) were purchased from MedChemExpress (Shanghai, China). Primary antibodies directed against GPX4 were obtained from ZenBio Science (Chengdu, China), and antibodies directed against PPAR-α, 14-3-3η, PTGS2, and β-actin were purchased from Proteintech (Chicago, IL, USA).

Adult male C57BL/6 mice were purchased from the Animal Center of Nanchang University (Nanchang, China). The experimental procedure followed the National Institutes of Health (NIH) guidelines and ARRIVE guidelines and was authorized by the Animal Experimentation Ethics Committee of Nanchang University (No. NCULAE-20221031133).

### In vitro experiments

#### Culture of H9c2 cells and construction of an anoxia/reoxygenation (A/R) injury model

H9c2 cells were obtained from the Cell Bank/Stem Cell Bank (Beijing, China), and were maintained in Dulbecco’s modified Eagle’s medium (DMEM) (Hyclone, GE Healthcare Life Sciences, Pittsburgh, PA, USA) supplemented with 10% fetal bovine serum (FBS) (Gibco, Thermo Fisher Scientific, Waltham, MA, USA) at 37 °C under the following conditions 95% O_2_, 5% CO_2_, 37 °C.

As presented in a previous study^21^, an in vitro MIRI model (anoxia/reoxygenation injury model) was established as follows: First, H9c2 cardiomyocytes were cultured in anoxia lipid (CaCl_2_ 1.0 mM, HEPES 20 mM, KCl 10 mM, MgSO_4_ 1.2 mM, NaCl 98.5 mM, NaH_2_PO_4_ 0.9 mM, NaHCO_3_ 6 mM, and sodium lactate 40 mM, pH 6.8) at 95% N_2_ and 5% CO_2_ for 3 h. Subsequently, reoxygenation lipid (CaCl_2_ 1.0 mM, glucose 5.5 mM, HEPES 20 mM, KCl 5 mM, MgSO_4_ 1.2 mM, NaCl 129.5 mM, NaH_2_PO_4_ 0.9 mM, and NaHCO_3_ 20 mM, pH 7.4) was applied to mimic reperfusion, and H9c2 cardiomyocytes were cultured in 95% O_2_ and 5% CO_2_ for 2 h (normal conditions).

#### Adenovirus transduction

pAd/14-3-3η-shRNA and pAd/NC-shRNA (Gene Pharma Co., Ltd, Suzhou, China) were transduced into H9c2 cells and cultured in normal conditions. 24–48 h later, when the transfection efficiency reached about 85%^22^, the following experimental procedure were conducted.

#### H9c2 cardiomyocytes grouping and treatment with reagents.

H9c2 cardiomyocytes were divided into seven groups: (1) Control group: H9c2 cells were incubated in normal conditions; (2) A/R group: H9c2 cells exposed to A/R treatment; (3) GW6471 + A/R group: H9c2 cells treated with 1 µM GW6471 for 24 h before A/R injury; (4) GW7647 + A/R group: H9c2 cells treated with 1 µM GW7647 for 24 h before A/R injury; (5) Fer-1 + A/R group: H9c2 cells treated with 5 µM Fer-1 for 2 h before A/R injury; (6) GW7647 + pAd/14-3-3η-shRNA + A/R group: H9c2 cells transfected with pAd/14-3-3η-shRNA for 48 h and treated with GW7647 for 24 h before A/R injury; (7) pAd/NC-shRNA + GW7647 + A/R group: H9c2 cardiomyocytes treated with pAd/NC-shRNA and GW7647 for 48 h and 24 h before A/R injury.

#### Assessment of cell viability and cytotoxicity

The cell viability and level of lactate dehydrogenase (LDH) in H9c2 cardiomyocytes after different treatments were assessed via the Cell Counting Kit-8 assay (CCK-8) (Good Laboratory Practice Bioscience, Montclair, CA, USA) and the LDH assay kit (Beyotime, Shanghai, China) based on the manufacturer’s protocols.

#### Assay of caspase-3 activities, lipid peroxidation, and total iron levels

The contents of Malondialdehyde (MDA), total iron, GSH, and glutathione disulfide (GSSG), and the activity of caspase-3 in H9c2 cells were detected following the protocols provided with the MDA assay kit, GSH and GSSG assay kit, caspase-3 activity assay kit (Beyotime, Shanghai, China), and total iron assay kit (Nanjing Jiancheng Bioengineering Institute, Nanjing, China).

#### Detection of ferrous iron

Ferrous iron was detected using FerroOrange (Dojindo, Tokyo, Japan) according to the manufacturer’s instructions. In brief, after treatment, H9c2 cardiomyocytes were cultured with,1 µM FerroOrange for 30 min at 37 °C in the dark. Then, the level of ferrous iron was evaluated using an Olympus IX 73 microscope (Olympus, Tokyo, Japan).

#### Measurement of Adenosine 5′-triphosphate (ATP) generation

The level of ATP in H9c2 cardiomyocyte homogenates was detected using ATP assay kits (Nanjing Jiancheng Bioengineering Institute, Nanjing, China) based on the manufacturer’s instructions.

#### Mitochondrial membrane potential assay

The mitochondrial membrane potential (MMP) was assessed through the JC-1 MMP detection kit (BestBio, Shanghai, China) according to the manufacturer’s instructions. In brief, after treatment, H9c2 cells were cultured with JC-1 for 30 min at 37 °C in the dark. Then, fluorescence was evaluated using an Olympus IX 73 microscope (Olympus, Tokyo, Japan).

#### Flow Cytometry assay

Mitochondrial permeability transition pore (mPTP), cell apoptosis, and the levels of ROS and lipid ROS were determined by the mPTP detection kit (BestBio, Shanghai, China), Annexin V-FITC apoptosis detection Kit (BestBio, Shanghai, China), ROS assay kit (Beyotime, Shanghai, China), and lipid ROS assay kit (GLPBIO, Montclair, CA, USA) according to the protocol provided with the corresponding assay kit. After treatment, H9c2 cardiomyocytes were incubated with BbcellProbe M61, C11-BODIPY581/591, 2,7-dichlorodihydrofluorescein diacetate (DCFH-DA), Annexin V-FITC, or propidium iodide for 30 or 15 min at 37 °C or 4 °C in the dark. Next, mPTP opening was detected using a Cytomics FC500 flow cytometer (Beckman Coulter, Brea, CA, USA) (excitation (Ex) = 488 nm, emission (Em) = 558 nm), apoptosis rate was determined using the same flow cytometer (Cytomics FC500, Ex = 488 nm, Em = 578 nm), the intracellular contents of ROS were assessed using the same flow cytometer (Cytomics FC500, Ex = 488 nm, Em = 525 nm). Moreover, the level of lipid ROS was determined by a flow cytometer (Cytomics FC500, Ex = 482 nm, Em = 590 nm).

#### Immunofluorescence assessment

After treatment, H9c2 cells were blocked with 1% bovine serum album in PBST for 2 h at 37 °C after being placed in 4% paraformaldehyde (PFA) for 15 min at 25 °C and permeabilized with 0.5% Triton X-100 in PBS for 20 min at 25 °C. Subsequently, H9c2 cardiomyocytes were incubated with an antibody directed against PPAR-α (ZenBio Science, Chengdu, China) (1: 50) at 4 °C for 12 h before being stained with a secondary antibody (Beyotime, Shanghai, China, 1: 600) for 1 h at 37 °C in the dark. Subsequently, nuclei were stained using 4′,6-diamidino-2-phenylindole (DAPI) at 25 °C for 10 min in the dark. Finally, fluorescence images were collected by an Eclipse C1 fluorescence microscope (Nikon, Tokyo, Japan).

#### PPAR-α/14-3-3η dual-luciferase reporter system

The 3′-UTR of 14-3-3η, which includes the binding site of PPAR-α, was cloned downstream of the dual-luciferase reporter gene assay in the psiCheck2 vector after amplification by overlapping PCR. In brief, H9c2 cells were transfected with psiCheck2-14-3-3η -promoter plasmids, pcDNA3.1-PPAR-α plasmids, and reporter plasmids for 48 h. Then, cells were assessed via the dual-luciferase reporter gene assay kit (RG-027, Beyotime, Nanjing, China).

### In vivo experiments

#### Construction of the MIRI model and experimental grouping

Mice were divided into four groups: (1) Sham group; (2) I/R group; (3) I/R + GW7647 group; and (4) I/R + GW7647 + Compound C11 group. Mice in the Sham and I/R group were injected with normal saline; Mice in I/R + GW7647 group and I/R + GW7647 + Compound C11 group were injected intragastrically (ig) with 3 mg/kg GW7647 or intraperitoneally (i.p) with 20 mg/kg Compound C11 daily for two weeks. After anesthetizing the mice with 1.5% isoflurane, mice were placed in the supine position, a left thoracotomy was conducted, and the pericardium was opened. The left anterior descending artery (LAD) was ligated with a 4–0 silk suture and the ends of the suture were passed by a short polyethylene tube to form a snare. The snare was clamped against the heart surface for ischemia and the snare was released for reperfusion. The sham group underwent the same procedure but without clamping of the LAD. Moreover, mice were analgesia with meloxicam (5 mg/kg). To mimic MIRI in vivo, mouse hearts were ischemic for 1 h and were reperfused for 24 h.

#### Assay of infarct size

Evans Blue and triphenyl tetrazolium chloride (TTC) double staining was conducted to determine infarct size. Briefly, the LAD was clamped following 24 h of reperfusion, after which 1.5 ml of 0.5% Evans blue dye was injected into the left ventricle. Next, hearts were cut into 1-mm cross-axis sections, and cultured with 2% TTC (Solarbio Life Sciences, Beijing, China) at 37 °C for 20 min after being frozen in liquid nitrogen for 30 s. Then, the tissue sections were placed in 4% PFA for 24 h and photographed.

#### Detection of LDH, CK-MB, and Fe^2+^ contents

After the mice underwent treatment, the serum samples were obtained after blood was collected and centrifuged for 10 min at 4 °C. Then, the contents of LDH, CK-MB, and Fe^2+^ in serum were determined according to the protocol provided with commercial detection kits (Jiancheng, Nanjing, China).

#### Echocardiography

After reperfusion, mice were anesthetized with 1.5% isoflurane, and cardiac function was examined using two-dimensional transthoracic echocardiography (Vevo2100 imaging system, VisualSonics Inc, Toronto, Canada).

#### Dihydroethidium (DHE) Staining

DHE staining was conducted following the manufacturer’s instructions. Briefly, 8-µm frozen sections of the left ventricle were cultured with DHE solution (Servicebio, Wuhan, China) at 37 °C for 1 h, then fluorescent images were acquired by a Nikon fluorescent microscope (Tokyo, Japan).

#### TUNEL staining

Following different treatments, cardiac specimens were gathered, fixed with 4% PFA, and embedded in optical coherence tomography (OCT) after dehydration with sucrose. Then, cardiac specimens were cut into 3-µm-thick sections and TUNEL staining was performed according to the protocol provided with the One-Step TUNEL Apoptosis Assay Kit (Beyotime, Shanghai, China). Images of the staining were taken using a Nikon fluorescent microscope (Nikon, Tokyo, Japan).

#### Histopathological observation

Briefly, mouse hearts were fixed in 4% PFA and cut into 5-µm cross-axis sections after being embedded in paraffin. Then the sections were cultured with hematoxylin and eosin (H&E) and evaluated using an optical microscopy (Olympus, Tokyo, Japan).

#### Assessment of MDA and total iron concentration

The levels of MDA and total iron in left ventricular tissue homogenates were determined based on the protocols of the MDA assay kit (Beyotime, Shanghai, China) and total iron assay kit (Nanjing Jiancheng Bioengineering Institute, Nanjing, China).

#### Mitochondrial ultrastructural assessment

Following different treatments, fresh samples of left ventricle papillary muscle or H9c2 cells were quickly collected and placed in 2% glutaraldehyde for 2 h. Then, transmission electron microscopy (TEM) (Hitachi 7800, Tokyo, Japan) was employed to detect the level of mitochondrial damage after washing, dehydration, embedding, sectioning, and staining. Moreover, mitochondrial ultrastructural damage was determined using the Flameng score method^23^. Briefly, the Flameng score method scores mitochondria based on morphological changes, with a score of 0 for a normal structure, 1 for a normal structure and loss of granules, 2 for swollen mitochondria with clear matrix, 3 for broken mitochondrial cristae and the condensation of matrix, and 4 for broken mitochondrial cristae and loss of integrity of mitochondrial inner and outer membrane.

#### Western blot

After treatment, H9c2 cells and myocardial tissues were lysed in RIPA buffer with 1% PMSF (Beyotime, Shanghai, China), cultured at 4 °C for 15 min. Then, the BCA protein assay kit (Beyotime, Shanghai, China) was used to quantify the protein concentration. Per sample, a total of 30 µg protein was loaded onto a 10% sodium dodecyl sulfate–polyacrylamide gel (SDS-PAGE) and electrophoresis was performed. Proteins were transferred to polyvinylidene fluoride (PVDF) membranes, and membranes were blocked with 5% non-fat dry milk in TBST buffer at room temperature for 2 h. Subsequently, membranes were cultured with primary antibodies directed against PTGS2, GPX4, 14-3-3η, PPAR-α, and β-actin at 4 °C for 12 h. Subsequently, membranes were cultured with secondary antibodies (Beyotime, Shanghai, China) for 2 h at 25 °C. Finally, protein bands were visualized using an ultrahigh sensitivity ECL Kit (Beyotime, Shanghai, China).

### Statistical analysis

Statistical analyses were conducted using GraphPad Prism (La Jolla, CA, USA) and data are presented as the mean ± standard deviation (SD). Data were evaluated by one-way analysis of variance (ANOVA) and Dunnett’s or Turkey’s post hoc multiple comparisons test and p-value ≤ 0.05 was considered to be statistically significant.

### Ethics approval and consent to participate

All animal experiments were approved by the Animal Experimentation Ethics Committee of the Nanchang University (ethics number: NCULAE-20221031133).

## Results

### PPAR-α prevented ferroptosis in A/R-treated H9c2 cells

**Figure 1:**
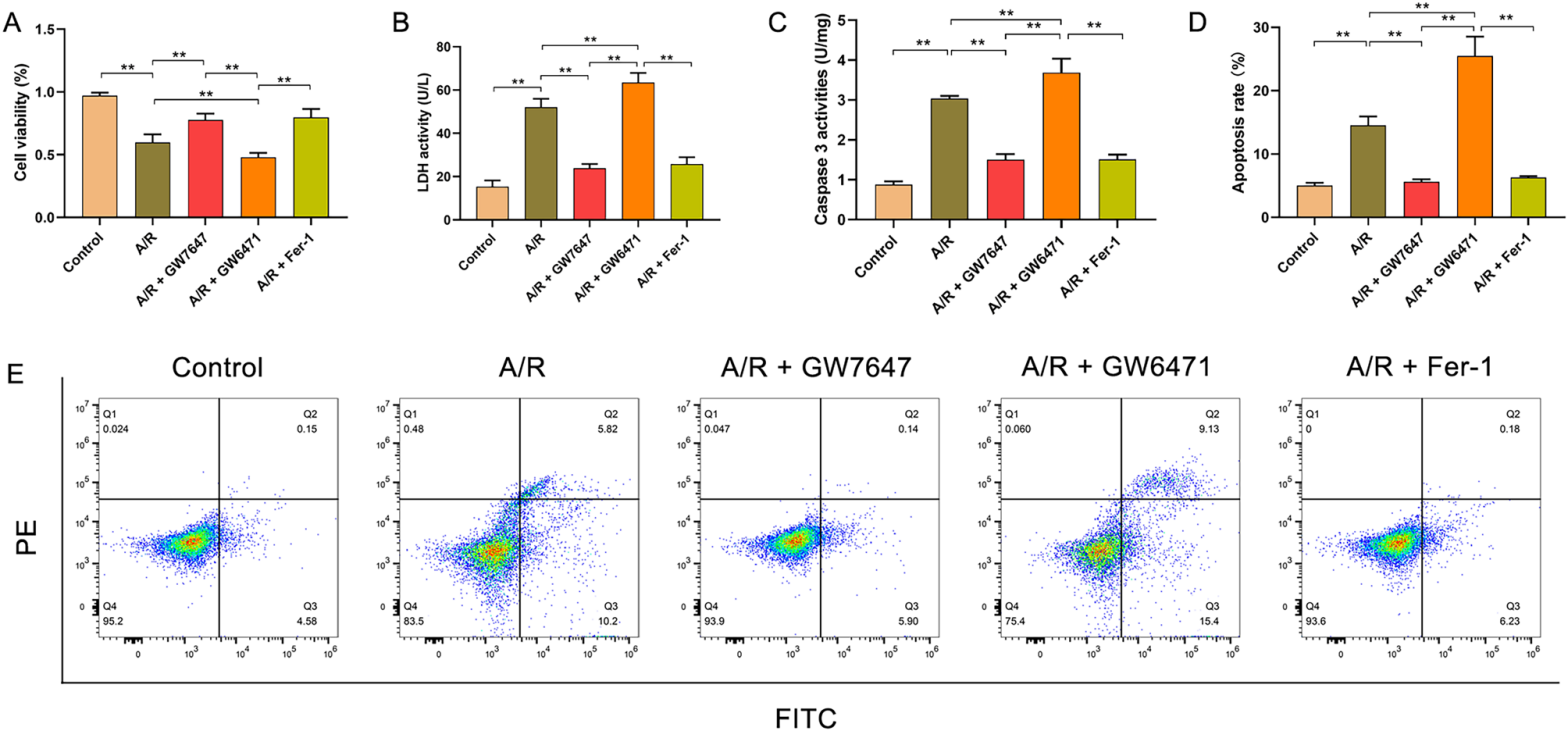
Ppar-α alleviated A/R injury in the H9c2 cells. (**A**) CCK-8 detected the cell viability in A/R-induced cells after GW7647, GW6471, or Fer-1 pretreatment. (**B**) the concentrations of LDH activities determined by corresponding quantitative kits in A/R-induced cells after GW7647, GW6471, or Fer-1 induced. (**C**) Caspase-3 activity was measured using a Caspase-3 quantitative kit in A/R-induced cells after GW7647, GW6471, or Fer-1 pretreatment. (**D**, **E**) Apoptotic rate measured by Annexin V-FITC/PI detected by flow cytometry. Data are expressed as the mean ± SD (n = 3). **P < 0.05.

To explore the role of PPAR-α on H9c2 cells treated with A/R, we detected cell viability, the LDH level, and cell apoptosis in A/R-induced H9c2 cardiomyocytes. Compared with the control group, the LDH level and cell viability were remarkably elevated/reduced in the A/R group (Fig. 1A,B). However, the cell viability was obviously induced and LDH levels were significantly decreased in the GW7647 + A/R group. Similarly, the apoptosis rate of H9c2 cells and the activities of caspase-3 in H9c2 cells were remarkably increased after A/R treatment, and which could be canceled by GW7647 (PPAR-α activator) pretreatment (Fig. 1C–E). However, these harmful effects of A/R injury could be promoted by GW6471 (PPAR-α inhibitor). Together, these results demonstrated that activation of PPAR-α could effectively protect H9c2 cardiomyocytes from A/R injury. Additionally, similar results were achieved with 5 µM of Fer-1 (ferroptosis inhibitor) in A/R-treated H9c2 cells, thereby indicating that the cardioprotective effects of PPAR-α activation might be associated with the attenuation of ferroptosis induced by A/R injury (Fig. 1A–E).

**Figure 2:**
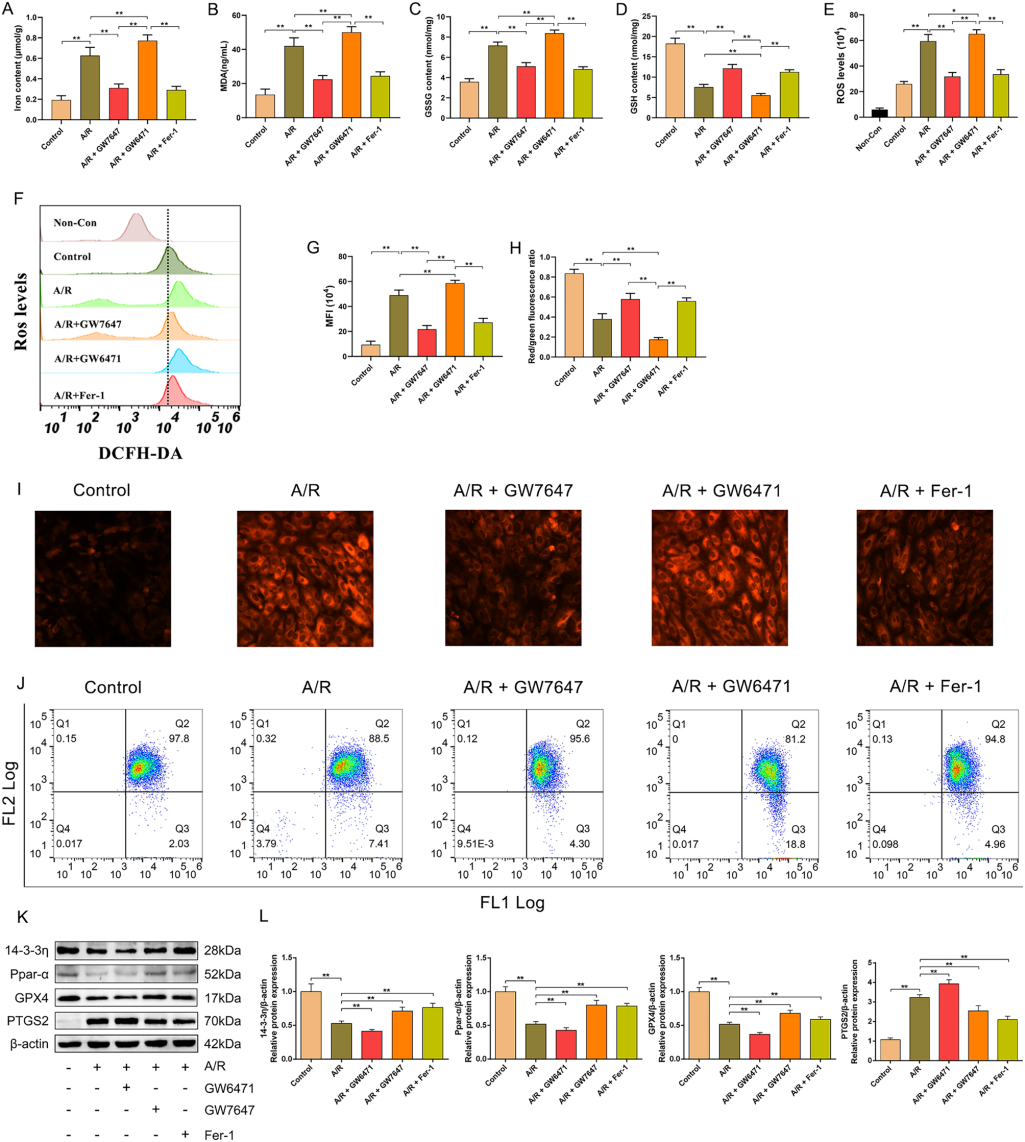
Ppar-α inhibits ferroptosis of A/R-induced H9c2 cells by downregulating 14-3-3η. The concentrations of (**A**) MDA, (**B**) total iron, and (**C**, **D**) GSH and GSSG were determined by corresponding quantitative kits in A/R-induced cells after GW7647, GW6471, or Fer-1 pretreatment. The levels of (**E**, **F**) ROS and (**G**, **I**) ferrous iron were measured by corresponding quantitative kits in A/R-induced cells after GW7647, GW6471, or Fer-1 induced (magnification, ×200). (**H**, **J**) The levels of Lipid ROS was assessed by C11-BODIPY staining. (**K**, **L**) the expression of 14-3-3η, Ppar-α, and ferroptosis-related proteins were detected by Western blot in A/R-induced cells after GW7647, GW6471, or Fer-1 pretreatment. Data are expressed as the mean ± SD (n = 3). **P < 0.05.

Recent studies have shown that iron overload and lipid peroxidation are hallmarks of ferroptosis^24,25^. Hence, experiments were conducted to confirm the role of PPAR-α activity on the levels of total iron, ferrous iron, lipid ROS, MDA, ROS, GSH, and GSSG in H9c2 cells treated with A/R. As shown in Fig. 2A–D, the intracellular levels of total iron, MDA, and GSSG were remarkably elevated and the level of GSH was reduced in A/R-induced H9c2 cardiomyocytes, while the effects of A/R injury could be remarkably abolished/promoted by GW7647 and GW6471 pretreatment. Consistently, the levels of ferrous iron, ROS, and lipid ROS were remarkably increased in the A/R group compared with the control group, and pretreatment with GW7647 or Fer-1 could effectively reduce the ferrous iron content. Additionally, the levels of ferrous iron were remarkably increased in the A/R + GW6471 group compared with the A/R group. (Fig. 2E–J). To further verify that activation of PPAR-α could effectively attenuate A/R-induced ferroptosis, the expression levels of PTGS2 and GPX4 (two molecular markers of ferroptosis) were evaluated. As shown in Fig. 2K,L, the expression level of PTGS2 was obviously induced and the protein level of GPX4 was reduced when H9c2 cells were treated with A/R. However, GW7647 or GW6471 pretreatment could effectively cancel/promote the harmful effects of A/R treatment. Combined, our results demonstrated that PPAR-α activation could obviously attenuate A/R injury via preventing ferroptosis. To confirm whether 14-3-3η participates in the anti-ferroptosis effects of PPAR-α, we determined the expression levels of PPAR-α and 14-3-3η. The results indicated that the expression levels of 14-3-3η and PPAR-α were remarkably decreased in the A/R group, and GW7647 or GW6471 pretreatment could effectively increase/decrease the reduced expression levels of PPAR-α and 14-3-3η (Fig. 2K,L), thus suggesting that the anti-ferroptosis effects of PPAR-α activation are related to the upregulation of 14-3-3η.

### PPAR-α prevented mitochondrial injury in A/R-induced H9c2 cells

**Figure 3:**
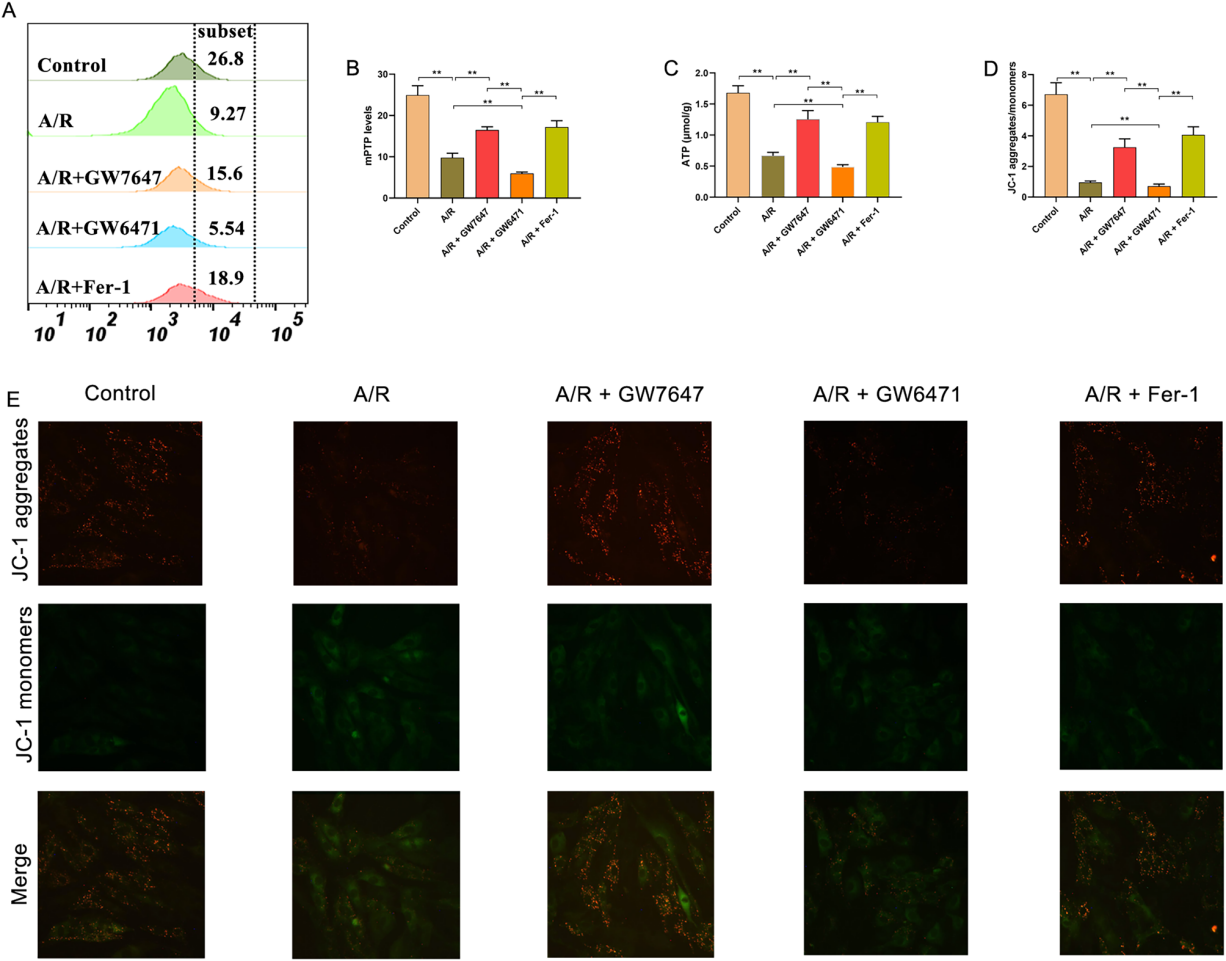
Ppar-α inhibits mitochondrial injury of A/R-induced H9c2 cells by downregulating 14-3-3η. (**A**) Fluorescent probe BBcellProbe M61 indicating mPTP opening was detected by flow cytometry. (**B**) Data summary of mPTP flow cytometry results. (**C**) The concentrations of ATP were determined by corresponding quantitative kits in A/R-induced cells after GW7647, GW6471, or Fer-1 induced. (**D**, **E**) MMP levels detected by JC-1 in H9c2 cells are indicated by the red/green fluorescence ratio. Data are expressed as the mean ± SD (n = 3). **P < 0.05.

Previous studies have reported that mitochondria play a key role in energy metabolism in cardiomyocytes, and participate in the pathological process of MIRI by regulating cell death, such as apoptosis, autophagy, and necrosis^26,27^. To confirm that activation of PPAR-α could effectively attenuate A/R-induced ferroptosis through preventing mitochondrial injury, the levels of MMP and ATP and the extent of mPTP opening were evaluated by flow cytometry. As shown in Fig. 3A,B, the level of MMP was decreased in H9c2 cells treated with A/R, which was significantly prevented by GW7647 or Fer-1 pretreatment. As shown in Fig. 3C, the ATP content in H9c2 cells treated with A/R was markedly lower than that in control cells, and GW7647 and Fer-1 pretreatment remarkably prevented the decrease in ATP. It is well established that MMP reduction and intracellular ROS accumulation leads to mPTP opening^28^. Consistently, our results showed that A/R injury could remarkably induce opening of the mPTP, which could be prevented via GW7647 or Fer-1 pretreatment (Fig. 3D,E). Interestingly, by treatment with GW6471, these harmful effects of A/R injury were promoted. Thus, these results indicated that activation of PPAR-α could effectively attenuate A/R-induced mitochondrial injury.

### PPAR-α alleviates ferroptosis and mitochondrial injury in A/R-treated H9c2 cells by regulating 14-3-3η

**Figure 4:**
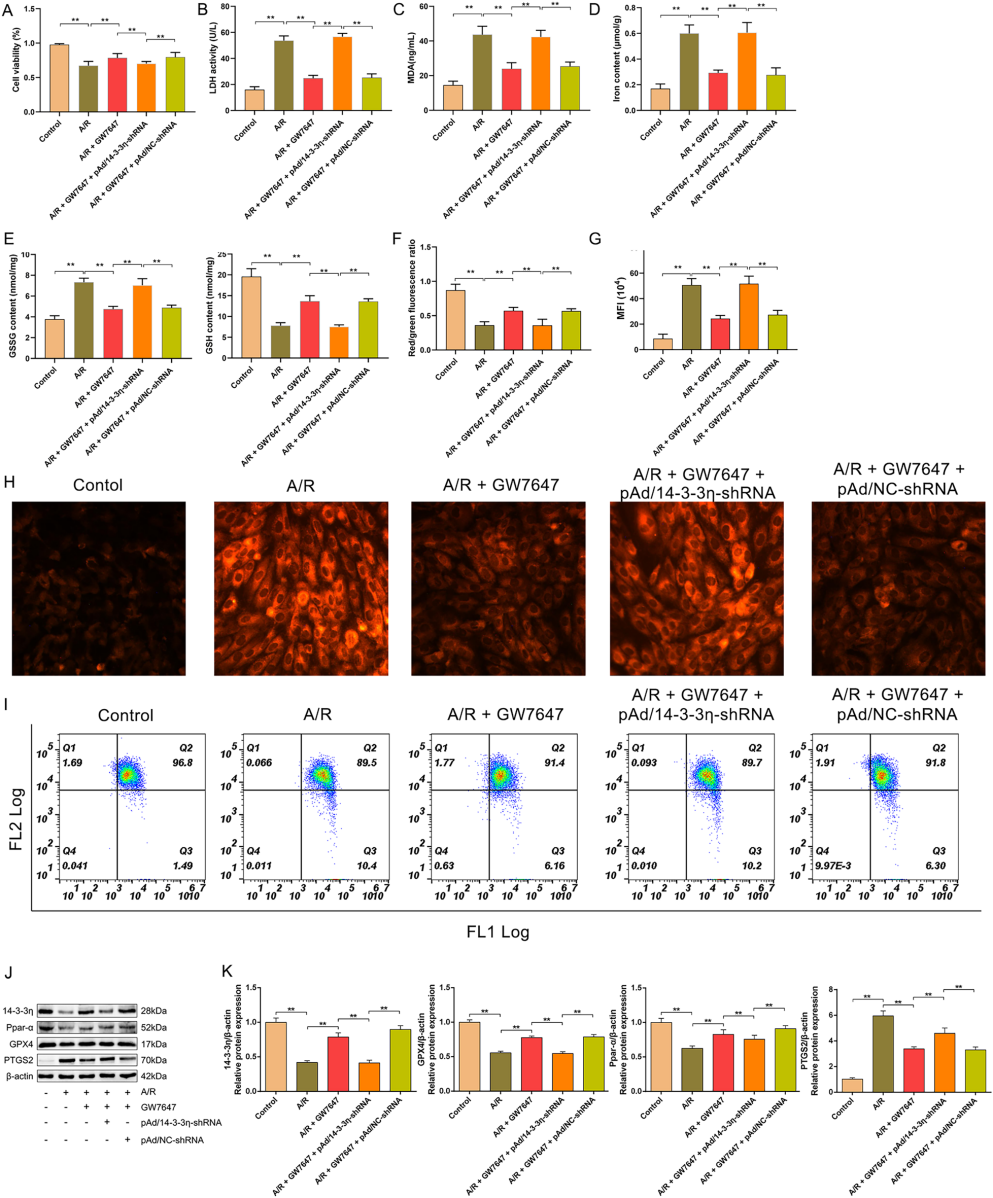
Ppar-α inhibits ferroptosis of A/R-induced H9c2 cells by mediating 14-3-3η. (**A**) CCK-8 detected the cell viability in A/R-induced H9c2 cells after GW7647, pAd/14-3-3η-shRNA, and pAd/NC-shRNA pretreatment. The concentrations of (**B**) LDH activities, (**C**) MDA, (**D**) total iron, and (**E**) GSH, GSSG, and GSH/GSSG were determined by corresponding quantitative kits in A/R-induced cells after GW7647, pAd/14-3-3η -shRNA, and pAd/NC-shRNA induced. (**F**, **H**) the concentrations of ferrous iron were measured by corresponding quantitative kits in A/R-induced cells after GW7647, pAd/14-3-3η-shRNA, and pAd/NC-shRNA pretreatment (magnification, ×200). (**G**, **I**) The levels of Lipid ROS was assessed by C11-BODIPY staining. (**J**, **K**) the expression of 14-3-3η, Ppar-α, and ferroptosis-related proteins were detected by Western blot in A/R-induced cells after GW7647, pAd/14-3-3η-shRNA, and pAd/NC-shRNA induced. Data are expressed as the mean ± SD (n = 3). **P < 0.05.

To elucidate whether, under MIRI injury, the PPAR-α/14-3-3η pathway participated in activating ferroptosis, the LDH activity and cell viability in H9c2 cells of each group were evaluated next. The data showed that A/R treatment could significantly decrease/increase cell viability/LDH activity, which could be increased/decreased by GW7647. Furthermore, pAd/14-3-3η-shRNA can cancel the protection of GW7647 (Fig. 4A,B). Our results also showed that compared with the control group, the contents of ferrous iron, lipid ROS, total iron, MDA, ROS, and GSSG were increased and the level of GSH was decreased in the A/R group. Interestingly, pretreatment with GW7647 remarkably abolished the harmful effects of A/R injury on the levels of ferrous iron, lipid ROS, total iron, MDA, GSH, and GSSG. Additionally, the protection of GW7647 was remarkably blocked by pAd/14-3-3η-shRNA (Fig. 4C–I). To further confirm whether PPAR-α activation attenuated ferroptosis through upregulating 14-3-3η in A/R-treated H9c2 cardiomyocytes, the protein levels of PTGS2, GPX4, PPAR-α, and 14-3-3η were evaluated. As shown in Fig. 4J,K, in H9c2 cardiomyocytes pretreated with GW7647, the expression levels of PTGS2/GPX4 were lower/higher than those in A/R-treated H9c2 cells. Additionally, the changed expression levels of PTGS2 and GPX4 were canceled by treatment with pAd/14-3-3η-shRNA. Moreover, the protein levels of 14-3-3η and PPAR-α were reduced in A/R-induced H9c2 cells, and GW7647 pretreatment could induce the levels of 14-3-3η and PPAR-α in H9c2 cells treated with A/R. However, the regulatory effects of GW7647 on PPAR-α could not be canceled after the addition of pAd/14-3-3η-shRNA (Fig. 4A–K). Taken together, these results suggested that activation of the PPAR-α/14-3-3η pathway could effectively attenuate A/R injury via preventing ferroptosis.

**Figure 5:**
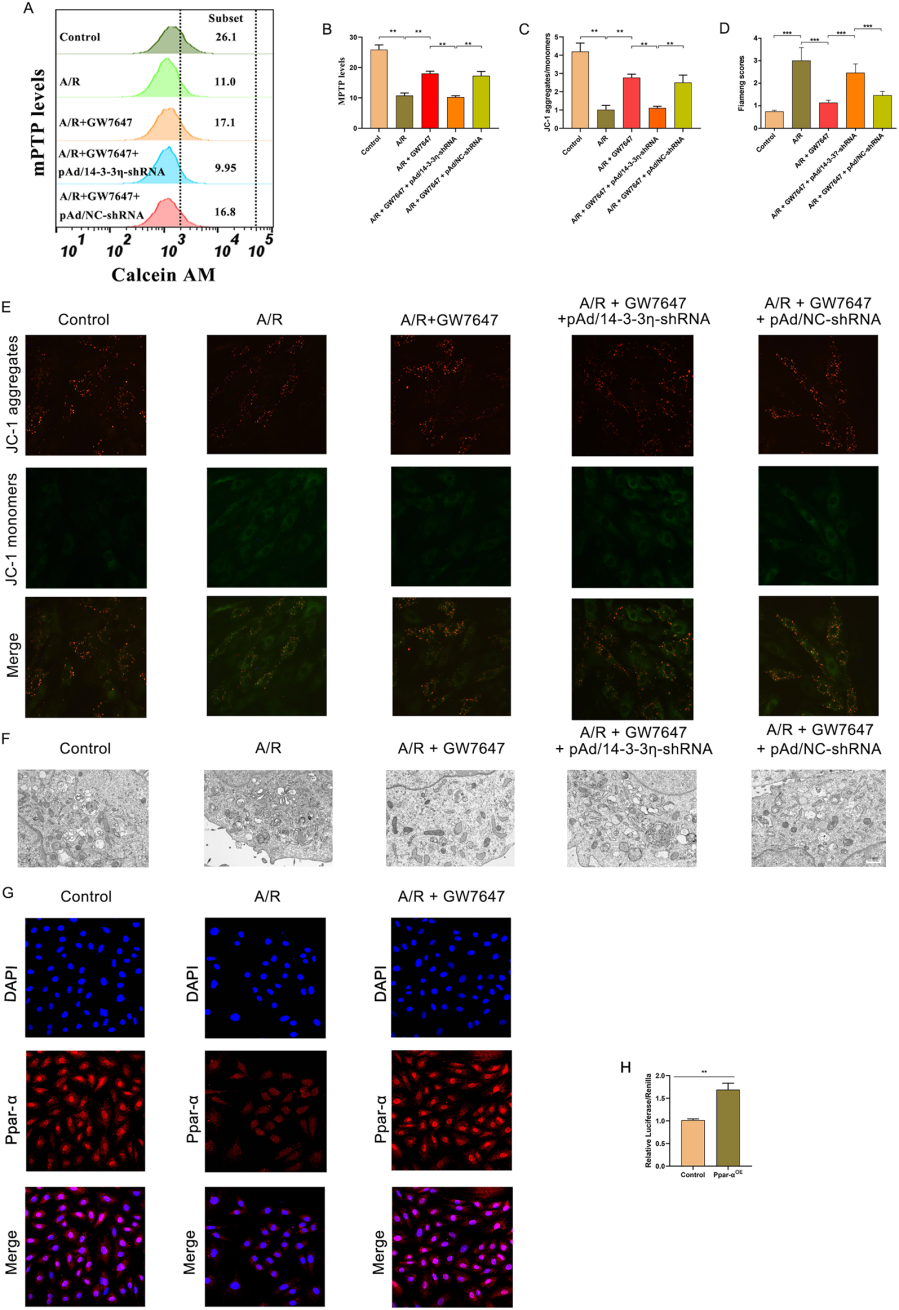
Ppar-α inhibits A/R-induced mitochondrial damage in H9c2 cardiomyocytes via modulating 14-3-3η. (**A**) Fluorescent probe BBcellProbe M61 indicating mPTP opening was detected by flow cytometry. (**B**) Data summary of mPTP flow cytometry results. (**C**, **E**) MMP levels detected by JC-1 in H9c2 cells are indicated by the red/green fluorescence ratio. (**D**) the Fiameng scores of H9c2 cells. (**F**) Transmission electron microscope images (magnification, ×6000). (**G**) Confocal immunofluorescence images showing Ppar-α translocation from the cytoplasm to nucleus after GW7647 pretreatment. (**H**) Luciferase results indicating the interaction between Ppar-α and 14-3-3η. Data are expressed as the mean ± SD (n = 3). **P < 0.05.

In a recent study, it was reported that mitochondria are one of the potential downstream target organelles of 14-3-3η^29^. Therefore, we also determined MMP levels, mPTP openness, and morphological changes in mitochondria. As shown in Fig. 5A,B, the levels of MMP in the A/R group were remarkably reduced compared to the control group, and the harmful effects of A/R injury on MMP could be reversed by GW7647 pretreatment. Simultaneously, A/R injury could remarkably induce opening of the mPTP, and such change was prevented by GW7647 (Fig. 5C,E). Moreover, TEM analysis suggested that in H9c2 cells induced by A/R, mitochondria were obviously deformed, cristae were reduced, and Fiameng scores were markedly induced, whereas GW7647 effectively prevented such morphological changes in mitochondria. Moreover, pAd/14-3-3η-shRNA can effectively reverse the above-mentioned protective effects of PPAR-α on damaged mitochondria (Fig. 5D,F). Thus, these results showed that activation of the PPAR-α/14-3-3η pathway could alleviate A/R injury via preventing mitochondrial injury.

### PPAR-α induces 14-3-3η expression by directly binding to its promoter region

To confirm whether the anti-ferroptosis effects of PPAR-α activation are related to inducing 14-3-3η transcription via binding to its promoter region, protein levels of nuclear PPAR-α and cytoplasmic PPAR-α were evaluated by immunofluorescence. Our results showed that A/R treatment could significantly induce cytoplasmic PPAR-α levels and reduce nuclear PPAR-α levels compared with the controls. However, nuclear and cytoplasmic PPAR-α levels were remarkably increased/decreased in H9c2 cells pretreated with GW7647 (Fig. 5G). Furthermore, to elucidate whether PPAR-α could bind to the 14-3-3η promoter and whether this is functional, the 14-3-3η promoter was cloned for luciferase reporter activity assays, and the results showed that PPAR-α promoted 14-3-3η expression (Fig. 5H).

### PPAR-α/14-3-3η pathway attenuates I/R-induced myocardial injury in mice

**Figure 6:**
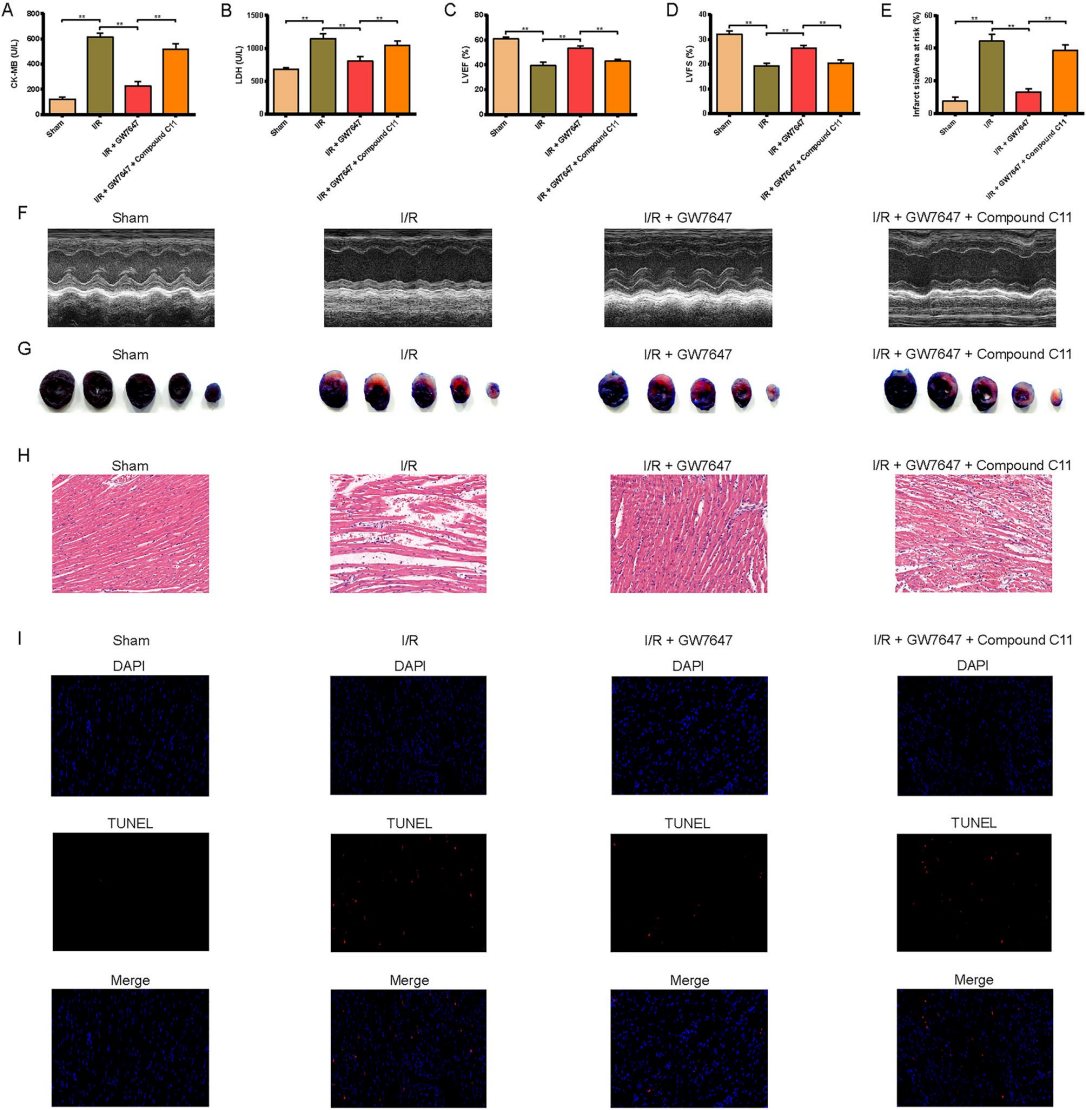
Ppar-α attenuated myocardial damage induced by MIRI via mediating 14-3-3η. (**A**, **B**) The activities of CK-MB and LDH in the serum of different group mice. Results of (**C**) LVEF and (**D**) LVFS. (**E**) Quantitative data for infarct size in mice hearts. (**F**) Representative images show the changes in cardiac structure detected by echocardiography in mice. (**G**) Representative images of infarct size were assessed using Evans blue/TTC double-staining. (**H**, **I**) Representative images of H&E staining and TUNEL staining for assessment of cardiac injury (magnification, ×200). Data are expressed as the mean ± SD (n = 3). **P < 0.05.

An in vivo MIRI model was established to assess the role of PPAR-α in I/R-treated mice, and GW7647 was used. As shown in Fig. 6A,B, the levels of CK-MB and LDH (two marker enzymes for myocardial injury) were remarkably elevated in the serum of I/R-treated mice. In addition, the results of echocardiography demonstrated that the left ventricular ejection fraction (LVEF) and left ventricular fraction shortening (LVFS) were remarkably reduced in hearts of I/R-induced mice (Fig. 6C,E, and F). Furthermore, infarct sizes in the I/R group were remarkably induced compared to the control group (Fig. 6E,G). Thus, these results show that the cardiac function of mice was obviously affected. However, pretreatment with GW7647 could effectively cancel these harmful effects of I/R injury (Fig. 6A–G), while pretreatment with Compound C11 could abolish the protective effects of GW7647.

Further morphological observation and evaluation suggested that after I/R-induced injury, mouse myocardial tissues exhibited an increased loss of cardiac fiber and an impaired cell arrangement, as indicated by H&E staining, and increased TUNEL-positive cardiomyocytes, as demonstrated by TUNEL staining (Fig. 6H,I). As expected, GW7647 could effectively cancel these changes in mice treated with I/R, while the effects of GW7647 were canceled by treatment with Compound C11.

### PPAR-α/14-3-3η pathway inhibits ferroptosis and mitochondrial injury in I/R-induced mice

**Figure 7:**
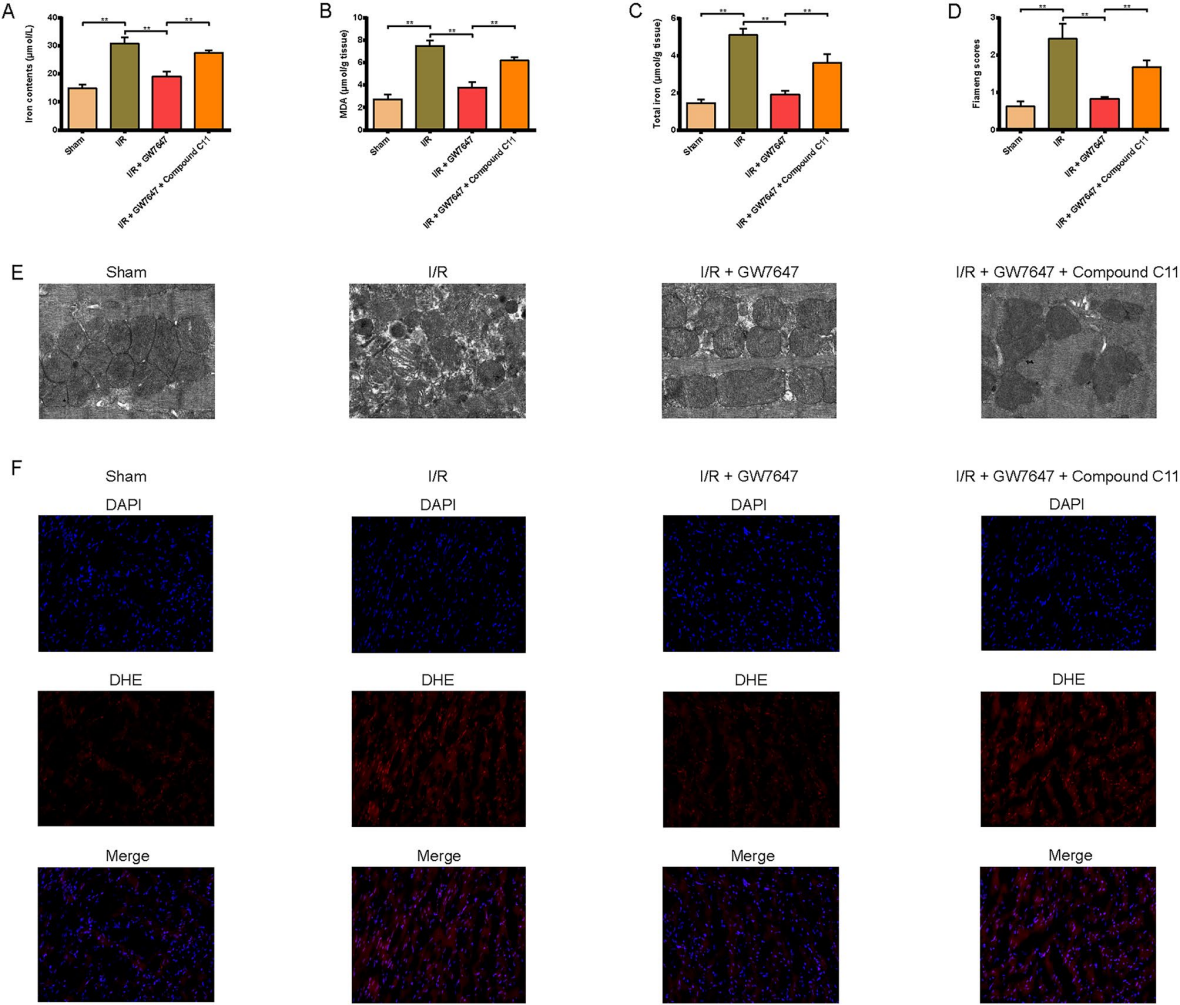
Effect of Ppar-α on parameters reflecting ferroptosis and mitochondrial damage in myocardial tissues of MIRI mice. (**A**) The iron contents of mouse serum in each treatment group. (**B**, **C**) MDA, total iron concentration levels in heart tissues. (**D**, **E**) Transmission electron microscopy images and the relative Fiameng scores of mouse myocardium (magnification, ×6000). (**F**) Representative images of DHE-stained mouse myocardium (magnification, ×200). Data are expressed as the mean ± SD (n = 3). **P < 0.05.

To further confirm the role of the PPAR-α/14-3-3η pathway under MIRI, the effects of GW7647 on ferroptosis, lipid peroxidation, and mitochondrial injury were assessed in the myocardium of mice induced by I/R injury. As shown in Fig. 7A–C and F, iron and MDA contents and the contents of ROS were markedly increased in the serum or myocardial tissues of mice treated with I/R, and the effects of I/R injury were significantly reversed by treatment with GW7647. Moreover, the following indicators: TEM observation, reflecting the mitochondrial injury in myocardial tissues of mice, were similar to the results of in vitro experiments (Fig. 7D–E). After pretreatment with Compound C11, the protective effects induced by GW7647 as described above was canceled. Combined, these results suggest that activation of the PPAR-α/14-3-3η pathway could effectively inhibit I/R injury via preventing mitochondrial injury and ferroptosis.

## Discussion

In this study, we explored the role of PPAR-α in MIRI and the associated mechanisms involved, thus suggesting a new therapeutic target and signaling pathway for mitigating MIRI. The major findings are as follows: (1) ferroptosis was significantly activated in MIRI in vivo and in vitro, and is related to mitochondrial dysfunction. (2) pretreatment with GW7647 (PPAR-α antagonist) and GW6471 (PPAR-α inhibitor) could effectively attenuate and promote MIRI by mediating ferroptosis and mitochondrial injury. (3) PPAR-α could alleviate MIRI via regulating 14-3-3η in vivo and in vitro. In general, a novel signaling pathway of PPAR-α/14-3-3η that enhances MIRI via activating ferroptosis was identified for the first time.

MIRI remarkably affects the prognosis of myocardial infarction, which is associated with complex molecular mechanisms and multiple types of RCD such as apoptosis, autophagy, ferroptosis, and pyroptosis^30^. Investigating the molecular mechanisms underlying MIRI is necessary to develop more effective treatments^31^. Ferroptosis is a newly discovered type of RCD induced by the deficiency of glutathione peroxidase 4 (GPX4) and is related to iron overload and lipid peroxidation. An important characteristic of ferroptosis is the excess generation of lipid peroxides on the cell membrane, which distinguishes ferroptosis from classical RCDs, such as necrosis, autophagy, and apoptosis processes^32,33^. Ferroptosis has been found to play a key role in various diseases, as well as MIRI. Wang et al. found that dexmedetomidine could significantly alleviate MIRI-induced ferroptosis through AMPK/GSK-3β/Nrf2^34^. Consistently, we also found in vivo and in vitro that ferroptosis was significantly triggered by MIRI based on various enzymatic, morphological, and functional indices.

The activation of PPAR-α is considered the most promising therapeutic strategy for inflammatory and metabolic diseases^35^. The genes encoding PPAR-α are located on human chromosome 22 and mouse chromosome 15^11^. The PPAR protein is organized into multiple structural domains. The N-terminal domain exerts activation function and determines the specificity of target genes. The DNA-binding domain contains two zinc fingers, allowing binding to DNA PPAR response elements. PPARs also interact extensively with other regulatory factors, thereby regulating the expression of PPAR-α in cardiomyocytes^36,37^. This finding highlights the indispensable regulatory role of PPAR-α in the heart. Previous studies have reported that PPAR-α could act as a ferroptosis inhibitor to prevent ferroptosis under metabolic dysfunction-associated fatty liver disease^38^. To explore the role of PPAR-α in MIRI-induced injury and ferroptosis, an in vivo and in vitro MIRI model was constructed and relevant biomarker tests were performed. Our results indicated that the cell viability was remarkably elevated by GW7647 pretreatment, accompanied by reduced apoptosis rates and LDH activities, and opposite results were achieved by adding GW6471, indicating that the activation of PPAR-α could effectively attenuate MIRI. Furthermore, to confirm whether PPAR-α could alleviate MIRI through inhibiting ferroptosis, multiple ferroptosis-related markers and the expression levels of PTGS2 and GPX4 were assessed. The results of this study revealed that GW7647 pretreatment could effectively reduce the intracellular levels of ferrous iron, lipid ROS, ROS, total iron, MDA, and GSSG, and induce the level of GSH in A/R-induced H9c2 cells, and opposite results were achieved after GW6471 pretreatment. Moreover, the cardioprotective effects of GW7647 were analog to the protection of Fer-1. These results indicate that the activation of PPAR-α could significantly attenuate MIRI by preventing ferroptosis. Although there are no definitive findings demonstrating the specific mechanism by which PPAR-α mediates ferroptosis in MIRI, studies have shown that reduced expression levels of PPAR-α in an immunoglobulin A nephropathy model could promote ferroptosis^39^. Furthermore, the PPAR-α agonist GW7647 has been shown to effectively inhibit ferroptosis, alleviate disruption of brain iron homeostasis in APP/PS1 mice, and mitigate neuronal inflammation and lipid peroxidation. Moreover, the activation of PPAR-α could attenuate ferroptosis, and the inactivation of PPAR-α can increase sensitivity to ferroptosis^16^. By combining the results of our experiments and the conclusions of the above-mentioned studies, we next explored the underlying mechanisms of the inhibitory effect of PPAR-α on ferroptosis.

The 14–3-3 family proteins mainly include seven known isoforms (β, γ, ε, η, ζ, σ, and θ), and have been shown to be involved in cell differentiation, transformation, autophagy, and apoptosis, by interacting with partner proteins^28^. Additionally, it has been reported that 14-3-3η plays a key role in myocardium injury induced by ischemia reperfusion^29,30^. Moreover, it is well established that 14-3-3η could assist in the translocation of PKCε or Bcl-2 from the cytoplasm to the mitochondria and protect cardiomyocytes from various injuries^31^. It has also been reported that knocking down 14-3-3η could eliminate the cardioprotective effects of Capsaicin, thus indirectly confirming the involvement of 14-3-3η in the process of MIRI^29^. While we have confirmed that activation of PPAR-α in the MIRI model can effectively inhibit ferroptosis, decrease infarct size, and improve heart function, the underlying molecular mechanisms by which PPAR-α mediates ferroptosis remain unclear. Wu et al. reported that Pparδ could upregulate 14–3-3ε expression by binding directly to the 14–3-3ε promoter^32^. Therefore, we hypothesized that PPAR-α could alleviate MIRI-induced ferroptosis by regulating 14-3-3η. To test this hypothesis, a PPAR-α antagonist and pAd/14-3-3η -shRNA were added to the experimental group. Our results indicated that despite the activation of PPAR-α, inhibitory effects of ferroptosis were not achieved due to the downregulation of 14-3-3η expression through adding pAd/14-3-3η-shRNA. Moreover, the results of luciferase reporter gene experiments demonstrated that PPAR-α could transcriptionally upregulate 14-3-3η expression, thereby indicating that the activation of PPAR-α could effectively alleviate MIRI-induced ferroptosis via regulating 14-3-3η.

Mitochondrial injury is a main sign of ischemia-treated cardiomyocytes, and reperfusion or reoxygenation could promote mitochondrial dysfunction, which affects ROS accumulation and hinders cardiomyocytes survival^40^. Interestingly, recent studies have reported that the occurrence of ferroptosis is often accompanied by mitochondrial dysfunction, which could also promote ferroptosis through leading to intracellular ROS accumulation^41^. It has been reported that overexpression of 14-3-3η could protect MIRI through maintaining mitochondrial homeostasis^28^. Taken together, our results showed that the activation of PPAR-α effectively attenuated mitochondrial injury in MIRI-induced cardiomyocytes via regulating 14-3-3η, as shown by increased MMP, mPTP closure, and intact mitochondria morphology.

Our findings confirmed that PPAR-α could alleviate MIRI-induced ferroptosis and mitochondrial injury via regulating 14-3-3η. However, our study has some limitations. Firstly, although our results indicated that PPAR-α can attenuate mitochondrial injury in cardiomyocytes treated with I/R or A/R via mediating 14-3-3η, the underlying mechanism of action by which 14-3-3η regulates mitochondria remains to be explored. Secondly, additional molecular biology experiments such as ChIP assay are necessary to demonstrate the direct binding of PPARα with the promoter region of 14-3-3η.

## Conclusion

In conclusion, our findings indicate that the activation of PPAR-α could effectively protect cardiomyocytes against MIRI via preventing ferroptosis and mitochondrial injury, which exerts its protection mainly via regulating 14-3-3η. Therefore, the PPAR-α/14-3-3η pathway may be an effective therapeutic target for MIRI.

### Supplementary Information

Supplementary Figures.

## Data Availability

The raw data supporting the conclusions of this article will be made available by the authors, without undue reservation, to any qualified researcher.
