# Supplementary figures and images for: Activation of PPAR-α attenuates myocardial ischemia/reperfusion injury by inhibiting ferroptosis and mitochondrial injury via upregulating 14-3-3η
Source: Sci Rep. 2024 Jul 2;14:15246. doi: 10.1038/s41598-024-64638-9 (PMC11219969; doi:10.1038/s41598-024-64638-9)

Figure 2K

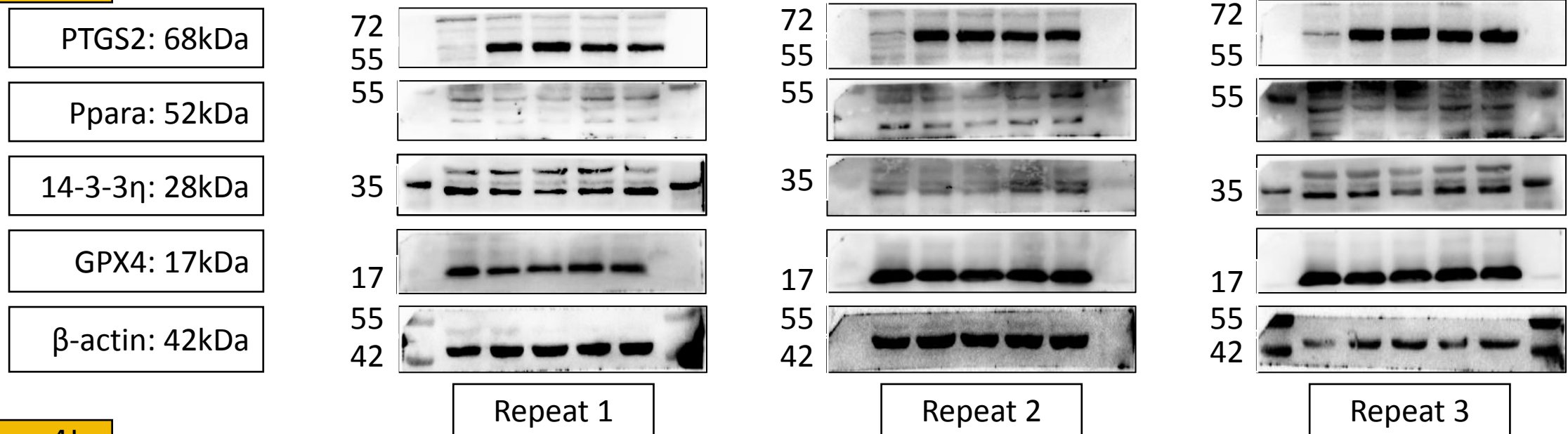

Figure 4J

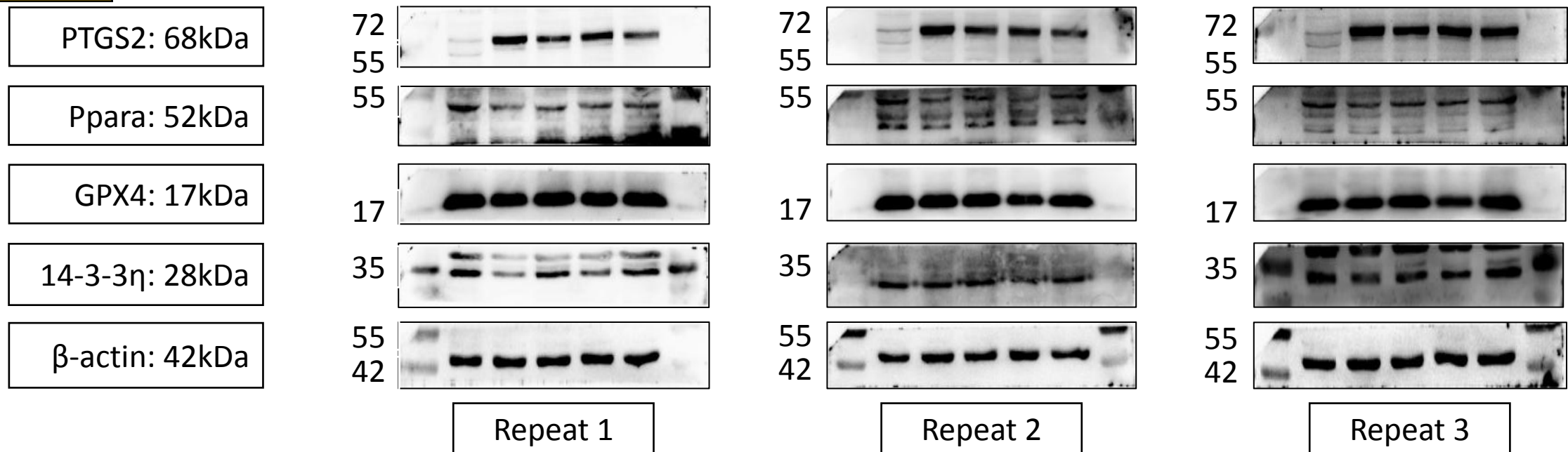

Supplement: Supplementary file 1 — Supplementary Figures. [file 41598_2024_64638_MOESM1_ESM.pdf]
